# Considerations for an Individual-Level Population Notification System for Pandemic Response: A Review and Prototype

**DOI:** 10.2196/19930

**Published:** 2020-06-05

**Authors:** Mohammad Nazmus Sakib, Zahid A Butt, Plinio Pelegrini Morita, Mark Oremus, Geoffrey T Fong, Peter A Hall

**Affiliations:** 1 School of Public Health and Health Systems University of Waterloo Waterloo, ON Canada; 2 Research Institute for Aging University of Waterloo and Schlegel Villages Waterloo, ON Canada; 3 Department of Systems Design Engineering University of Waterloo Waterloo, ON Canada; 4 eHealth Innovation Techna Institute University Health Network Toronto, ON Canada; 5 Institute of Health Policy, Management, and Evaluation Dalla Lana School of Public Health University of Toronto Toronto, ON Canada; 6 Department of Psychology University of Waterloo Waterloo, ON Canada; 7 Ontario Institute for Cancer Research Toronto, ON Canada

**Keywords:** pandemic, epidemic, notification system, hygiene, physical distancing, lockdown, mobile technology, COVID-19, coronavirus

## Abstract

The outbreak of the coronavirus disease (COVID-19), caused by severe acute respiratory syndrome coronavirus 2, spread worldwide after its emergence in China. Whether rich or poor, all nations are struggling to cope with this new global health crisis. The speed of the threat’s emergence and the quick response required from public health authorities and the public itself makes evident the need for a major reform in pandemic surveillance and notification systems. The development and implementation of a graded, individual-level pandemic notification system could be an effective tool to combat future threats of epidemics. This paper describes a prototype model of such a notification system and its potential advantages and challenges for implementation. Similar to other emergency alerts, this system would include a number of threat levels (level 1-5) with a higher level indicating increasing severity and intensity of safety measures (eg, level 1: general hygiene, level 2: enhanced hygiene, level 3: physical distancing, level 4: shelter in place, and level 5: lockdown). The notifications would be transmitted to cellular devices via text message (for lower threat levels) or push notification (for higher threat levels). The notification system would allow the public to be informed about the threat level in real time and act accordingly in an organized manner. New Zealand and the United Kingdom have recently launched similar alert systems designed to coordinate the ongoing COVID-19 pandemic response more efficiently. Implementing such a system, however, faces multiple challenges. Extensive preparation and coordination among all levels of government and relevant sectors are required. Additionally, such systems may be effective primarily in countries where there exists at least moderate trust in government. Advance and ongoing public education about the nature of the system and its steps would be an essential part of the system, such that all members of the public understand the meaning of each step in advance, similar to what has been established in systems for other emergency responses. This educational component is of utmost importance to minimize adverse public reaction and unintended consequences. The use of mass media and local communities could be considered where mobile phone penetration is low. The implementation of such a notification system would be more challenging in developing countries for several reasons, including inadequate technology, limited use of data plans, high population density, poverty, mistrust in government, and tendency to ignore or failure to understand the warning messages. Despite the challenges, an individual-level pandemic notification system could provide added benefits by giving an additional route for notification that would be complementary to existing platforms.

## Introduction

Despite a dramatic improvement in health care affordances and extensive public health measures around the world, the emergence and re-emergence of infectious pathogens and associated diseases have become a common phenomenon. The first 2 decades of the 21st century have already witnessed several major disease outbreaks such as severe acute respiratory syndrome (SARS) in 2003, with the most severe outbreak being the coronavirus disease (COVID-19) pandemic [1,2].

In December 2019, the outbreak of COVID-19, caused by a novel coronavirus named severe acute respiratory syndrome coronavirus 2 (SARS-CoV-2), was first reported in Wuhan, China [3,4]. Full-genome sequencing and phylogenic analysis revealed that SARS-CoV-2 is distinct from both severe acute respiratory syndrome–related coronavirus (SARS-CoV) and Middle East respiratory syndrome–related coronavirus (MERS-CoV) but closely linked [5]. Within a brief period, the disease has spread to virtually all regions of the world with a total of 6,112,902 confirmed cases and 369,593 deaths as of May 31, 2020 [6]. The World Health Organization (WHO) announced COVID-19 a “Public Health Emergency of International Concern” on January 30, 2020, and subsequently declared a pandemic on March 11, 2020 [7].

COVID-19 is primarily a respiratory illness and the majority of patients present with flu-like symptoms such as fever, cough, fatigue, shortness of breath, headache, and muscle pain; although, a significant minority experience different symptoms, some of which are highly distinctive (eg, ageusia, anosmia) [1,3,8,9]. Although most patients usually develop mild illnesses (81%), it can cause severe pneumonia, respiratory failure, and even death, particularly in individuals with an existing high-risk condition such as chronic obstructive pulmonary disease or diabetes [1,3,8,9]. It has been reported that about 14% of the patients develop a severe disease that requires hospitalization and oxygen support, and 5% require intensive care support [4,10]. Although the case fatality rate is expected to be around 0.5-1% [11,12], it is significantly higher among older adults and those with a high-risk condition [1,4,8]. Accordingly, countries with a higher older demographic are experiencing more critically ill patients and mortality in absolute terms [13,14].

In comparison to SARS and Middle East respiratory syndrome (MERS), which showed a mortality rate of 10% and 34%, respectively [15,16], COVID-19 appears to be less deadly. However, a sharp initial increase in the number of cases indicates that SARS-CoV-2 could be more contagious than the previously emerged coronaviruses [17]. The basic reproduction number (R_0_) of SARS-CoV-2, which indicates the average number of new cases generated from an infected person, is comparable to SARS [18]. Nevertheless, as a large number of patients develop a mild or asymptomatic form of the illness and may not seek medical advice, it greatly increases the risk of community transmission and larger outbreaks [19,20].

Overall, the emergence and re-emergence of highly infectious pathogens pose a substantial threat to human health. Air travel and tourism now allow for epidemics to quickly become pandemics [21]. In fact, the current pandemic was predicted and foreseen previously on multiple occasions [22]. The lessons from the ongoing global health crisis indicate that a substantial improvement is needed in our pandemic response, and this is possible by making use of mobile technology. The impact of future outbreaks can be mitigated through an improvement in pandemic notification systems by harnessing such technology.

## Existing Global Platforms for Infectious Disease Surveillance

The Program for Monitoring Emerging Diseases (ProMED; also known as ProMED-mail) is an internet-based, publicly available, and free of charge digital surveillance system dedicated for rapid collection and circulation of information on emerging infectious diseases and toxins that affect humans, animals, and plants [23,24]. This novel platform was founded in 1994 by the Federation of American Scientists and later became a part of the International Society for Infectious Diseases in 1999. ProMED has grown substantially since its establishment, and the number of subscribers increased to approximately 80,000, which represents every country in the world [23,24]. The ProMED system collects information from a variety of sources (eg, media reports, official reports, local observers); a multidisciplinary team of experts from different countries synthesizes the information, which is then disseminated to the subscribers directly by email and posted online on the ProMED website. The ProMED system continuously monitors emerging disease status and delivers updates as required in near real time. This platform was the first to report major disease outbreaks including SARS, MERS, Ebola, and Zika [23,24]. ProMED is now being used by a wide range of professionals and organizations including government officials, private organizations, researchers, physicians, and journalists.

Another global platform to address emerging public health issues is the Global Public Health Intelligence Network (GPHIN), developed by the Government of Canada in collaboration with the WHO [25]. Unlike ProMED, GPHIN is a fee-based service with subscription offers limited to only selected organizations involved in public health issues. GPHIN gathers information from global media sources (ie, Factiva and Al Bawaba) on a variety of public health topics [26,27], which is then filtered by an automated process for appropriateness and made accessible to the WHO, relevant government authorities, and subscribers. GPHIN reports constitute approximately 40% of the WHO’s early warning outbreak information [25]. Several other notable web-based disease surveillance systems are Influenzanet, the Global Outbreak Alert and Response Network, and Google Flu Trends [28-30].

Regarding the COVID-19 outbreak, both the ProMED and GPHIN systems delivered early notifications in late December 2019 of what turned out to be SARS-CoV-2, the virus causing COVID-19 [31]. Subsequently, SARS-CoV-2 spread worldwide, despite the presence of these early surveillance systems. It should be noted that these systems operate mostly at the level of government and public health authorities; therefore, an active engagement of the public throughout the process, which is a requirement for the preventive measures to be of utmost effectiveness, is an ongoing concern. Administration of an individual-level, population-wide alert system would be complementary to the existing platforms by enhancing public response and engagement.

## Rationale for an Individual-Level Pandemic Notification System

The COVID-19 pandemic emerged with such rapidity and lack of clarity on its transmissibility and severity that many governments implemented a cascade of changing, ad-hoc actions to address the situation [32,33]. In Canada, SARS and Ebola had primed the health care system to develop refined measures and protocols for dealing with cases, and to protect frontline health care workers [34,35]. Yet, there have been medical supply shortages and ongoing challenges to produce plans for managing incoming patients [36-40]. In the 8-week period leading up to the declaration of lockdown and social distancing measures, Canada, for example, moved from no travel restrictions and a declared low risk to the Canadian public to imposition of travel restrictions but not bans and selective banning of public gatherings [41]. These were then followed quickly by outright travel and gathering bans, coupled with stay-at-home orders [41]. This dynamic response played out across many countries around the world and continues as countries begin to lift restrictions. In the early stages, rapidly changing responses to the unfolding pandemic contributed to public confusion about expectations.

Throughout, the North American public has received communications from many sources of information, including those made by the Public Health Agency of Canada, the US Centers for Disease Control and Prevention, and regional counterparts. As we have witnessed with previous outbreaks, the COVID-19 pandemic has spawned a parallel, massive infodemic, leading to a surge of fabricated news and distorted evidence, which has increased the potential for health hazards while substantially diminishing the impact of valid information issued by health authorities about facts on all aspects of the virus, including origin, transmissibility, severity, and preventive measures [42-46]. Much like earlier trends, the presence of misinformation, rumors, and hoaxes about the novel coronavirus was also omnipresent throughout social media and the internet, which ultimately inflamed the crisis. To illustrate, there were claims about the origins of the virus (ie, bioterrorism and conspiracy theories) that led to xenophobia in many regions of the world [47,48]. Furthermore, some potentially dangerous rumors about miracle remedies spread in social media (ie, consumption of bleach, methanol, and disinfectant), which led to the deaths of people who were otherwise healthy and noninfected [49-51].

In addition, the media’s sensationalistic coverage of extreme events and reports without context (eg, bodies on the streets of Ecuador, Italian military hauling coffins) exacerbated the public reaction [52,53]. A spike in fear-driven panic buying of masks and other medical supplies occurred initially [54,55], ultimately contributing to a subsequent shortage on the front lines among hospital staff in some countries [36,37]. This suggests that some of the fear-driven responses among the public may have misdirected behavior in the early stages, in part due to confusion about what constituted an appropriate response to the level of threat at a given point in time.

Ideally, the public should be informed and advised by clear communications in a manner that can rise above the buzz and chaos of the plethora of information sources in the world. It must be evidence-based and driven by public health authorities who have the expertise to understand and interpret the rapidly growing scientific studies on the novel pathogen and can synthesize the material and communicate it in ways that the public can readily understand, and to provide advice in ways that the public can readily follow [43]. This may facilitate rule-based (better than emotion-based) responding among the population and diminish the unintended affect-driven responses that may be not desirable. Considering these matters, an individual-level, graded pandemic notification system, if implemented nationwide, could provide a significant benefit complementary to existing systems by ensuring a timely and coordinated response, transmitting notification directly to the general population via mobile devices. This will likely be important during multi-wave dynamic pandemics such as COVID-19 and during oscillation between response levels (ie, imposing and lifting restrictions).

## Prototype Model for an Individual-Level Pandemic Notification System

**Figure 1:**
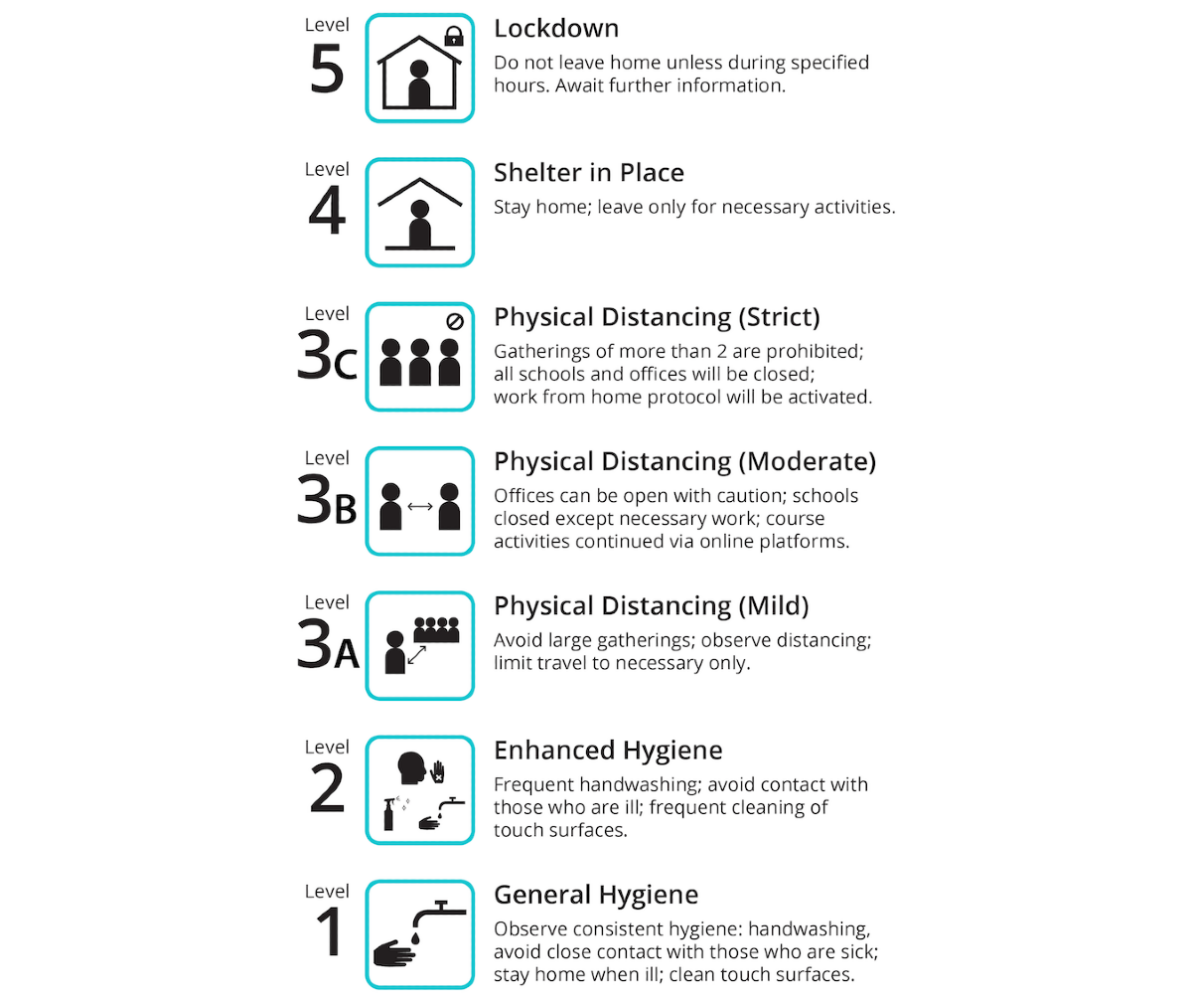
Sample icon and instruction set for a pandemic alert and response notification system.

As COVID-19 has spread worldwide with catastrophic effects on both economy and human life, there is a renewed consideration of an individual-level, general-purpose pandemic notification system responsive to outbreaks of an infectious illness. Such a system would make responses more universally clear and decipherable for the average member of the population. Much like a disaster or weather notification system, a pandemic notification system would be structured as a graded level of threats. Each level would ideally provide details on three aspects of the outbreak: information about the pathogen, level of threat, and behavioral directives (eg, hygiene, physical distancing). This system, known in advance by the public, would be used to prime the public in real time with respect to changes in threat level and, equally importantly, the expected responses. For example, if the system was operational now (Textbox 1, Figure 1), level 1 or 2 would provide information about SARS-CoV-2, the impending threat nationally and locally, and hygiene measures that need to be followed; threat level 3 would advise people toward physical distancing; level 4 would push stay-at-home orders; and level 5 would be more extreme measures such as a lockdown. Overall, the threat levels would be cumulative, and the public would follow all the precautionary measures outlined in the lower threat levels on top of the precautionary measures required by the current threat level put in place. Depending on the circumstances, the threat level could vary from region to region in a given country and could be switched as a given situation required. The mode of delivering the alert would be adjusted based on the intensity of the threat. For instance, the alert for a threat level of 1 or 2 may be disseminated by simple text messaging or passive information observed only for those who look at the relevant app or website source, but the alert for threat levels 3, 4, and 5 could be push notifications directly to the mobile phones of the entire population, similar to an Amber Alert (an Amber Alert is an emergency response system that provides immediate and up-to-date information to the public about a recently missing or abducted person) in Canada and other countries.

**Sample operational definitions and guidelines for a graded pandemic notification system. The threat levels are cumulative. Level 3A and above must include Enhanced Hygiene by default.**

**Level 5: lockdown**

This is the extreme level of precautionary measure, which requires a particular neighborhood to completely shut down all necessary and nonessential outdoor activities by the residents, with the exception of only law enforcement agencies, health care professionals, and personnel involved with essential services. Residents will contact the authority for emergency necessity and can go outside only with authorization. It is warranted when a large disease outbreak is confirmed in close vicinity, and the residents are exposed to severe risk of disease transmission.

**Level 4: shelter in place**

This is a high level of precautionary measure, which requires a particular neighborhood to completely shut down all nonessential outdoor activities by the residents. People can go outside only for necessary activities, which include but are not limited to buying food, getting medication, and essential in-person doctor’s appointments. It is warranted when a large disease outbreak is confirmed in close vicinity, and the residents are exposed to a high risk of disease transmission.

**Level 3C: physical distancing (strict)**

This is a high level of precautionary measure, which requires a particular neighborhood to follow strict physical distancing guidelines. Citizens must maintain a specific distance from each other while outdoors, with the exception of family members or individuals living in the same household. Any gathering of more than 2 people is prohibited, and noncompliance is subject to legal sanction. All schools and offices will be closed, and work from home protocol will be activated. It is warranted when a disease outbreak is confirmed in close vicinity, and the residents are exposed to a significant risk of disease transmission.

**Level 3B: physical distancing (moderate)**

This is a moderate level of precautionary measure, which requires a particular neighborhood to follow moderate physical distancing guidelines. Citizens must avoid large gatherings of a specific number (to be determined by the context of the locality). Offices can be open with caution and maintaining physical distancing guidelines. Schools will be closed except for the necessary activities. Course activities will be continued through online platforms. It is warranted when a disease outbreak is confirmed in close vicinity, and the residents are exposed to a significant risk of disease transmission, but the risk is lower than the Level 3C (to be determined by the number of cases, disease trend, and other vital information of the respective locality and surroundings).

**Level 3A: physical distancing (mild)**

This is a moderate level of precautionary measure, which requires a particular neighborhood to follow mild physical distancing guidelines. Citizens must avoid large gatherings of a specific number (to be determined by the context of the locality). Offices and schools can remain open with caution and maintaining adequate physical distancing guidelines. It is warranted when a disease outbreak is confirmed in close vicinity, and the residents are exposed to a significant risk of disease transmission, but the risk is lower than the level 3B (to be determined by the number of cases, disease trend, and other vital information of the respective locality and surroundings).

**Level 2: enhanced hygiene**

This is a mild level of precautionary measure, which requires a particular neighborhood to follow enhanced hygiene guidelines that include but are not limited to frequent handwashing, wearing masks where applicable, avoiding contact with people who are ill, and cleaning touched surfaces. It is warranted when a disease outbreak is suspected in close vicinity, and the residents are exposed to a general risk of disease transmission.

**Level 1: general hygiene**

This is a low level of precautionary measure, which requires a particular neighborhood to follow general hygiene guidelines that include but are not limited to observing consistent hygiene, handwashing, avoiding contact with people who are sick, staying home when ill, and cleaning touched surfaces. It is warranted when a disease outbreak is suspected in close vicinity, and the residents are exposed to a low risk of disease transmission.

Figure 1 presents an example of a symbol and number-coded system that is easy to remember and interpret by members of the public, even among those with limited literacy. Features include a hierarchical arrangement of threat levels and associated responses, the latter including links to more detailed information as necessary. If this were a universal standard within a given country, health care settings, workplaces, and educational institutions would also have a common understanding with employees, students, parents, and others on a moment-to-moment basis as to what is expected. Similar approaches have been widely tested and implemented in other domains, such as Defense Readiness Condition alert states [56] and travel advisories [57]. The integration of public education efforts along with drills or simulations during the time between threats would be useful to ensure that all have some level of recent familiarity with procedures and aspects of their implementation. Each of these settings could also be encouraged to include a response plan for their own institution, such that institutional protocols are worked out in advance.

If such a platform is available in a country capable of transmitting notification directly to the population through mobile messaging, it would improve the clarity of communication about both the changing threat level (which, in the case of an emerging pandemic, can be day-to-day) and what the expected steps are at each of these threat levels, as well as data-driven tools for helping public health officials to plan and monitor interventions. This could in turn be linked to reliable website information and data visualization for the public. The technology necessary for implementing these solutions is widely available and can easily be used to deliver the proposed services. However, major challenges lie in the privacy and governance of such platforms, requiring the guidance of a team of expert researchers in public health, privacy, and public policy to facilitate the implementation of such a solution.

Expectations on the level of everyday citizens, employers, and health care professionals could be aligned more easily by proactive creation of such a system, coupled with public education as to how to interpret and act on it. This latter facet is critically important—public education about the meaning of each level and the expected sequence of actions to take in response will reduce fear by making the response more obvious when the signal comes.

The public education and infrastructure investment for a pandemic notification system may require considerable funding; however, it could provide a long-term benefit to handle future pandemics once developed. Compared to SARS and MERS, COVID-19 will be costly to some countries due to delayed response and inefficient implementation of the recommendations [58,59]. A notification system would mitigate the initial confusion and improve implementational efficiency, which could, therefore, be economical in the long run.

## Prospects of Pandemic Notification Systems in Developed Countries

Developed countries have long-standing experience operating different alert systems successfully, such as the Emergency Alert System, Amber Alert, and Alert Ready. However, existing alert systems have been criticized for being inefficient for a myriad of reasons, such as the execution of false alarms, nonresponse, uneven distribution, network disruption, inadequate technology, and inappropriate timing [60-62]. Despite that, the necessity of emergency notification systems is indisputable, as a substantial number of lives could be saved and casualties could be prevented in the event of natural and human-made disasters [63,64].

Although the COVID-19 pandemic has spread pervasively, numerous strategies have been adopted by developed countries to communicate with the public about the threat and preventive measures undertaken. Some countries have excelled in their battle against COVID-19 because of prompt, coordinated, and organized actions. New Zealand, so far, is leading the battle against COVID-19 [65]. From the beginning, New Zealand has adopted the strategy of eliminating the virus rather than just containing it by imposing a national lockdown and other stringent measures [66]. In addition, a multilevel COVID-19 alert system, similar to terror alert, has been launched by the government of New Zealand to counter the ongoing crisis. This system comprises of 4 levels of alerts based on the impending risks of disease transmission: level 1“Prepare,” level 2 “Reduce,” level 3 “Restrict,” and level 4 “Lockdown” [67]. A higher level indicates an increased risk of disease transmission, therefore, associated with more stringent measures and restrictions. Each level has a risk assessment coupled with a range of public health and social measures expected to be followed by the residents. This is an organized and efficient way to communicate with the public about day-to-day restrictions in effect. New Zealand’s strategy appears to be working, as only 1504 confirmed cases and 22 deaths have been reported as of May 31, 2020, [68] and the transmissibility has been reduced to below outbreak threshold [65].

The United Kingdom has recently launched a five-tier coronavirus alert system to handle the pandemic situation more efficiently [69,70]. In this alert system, the threat levels of SARS-CoV-2 have been ranked on a scale of 1-5 with color coding for easy comprehension of the threat for the public: level 1 “Low” (green), level 2 “Moderate,” level 3 “Substantial” (orange), level 4 “Severe,” and level 5 “Critical” (red) [69,70]. The threat levels range from an absence of the virus corresponding to level 1 to a rapid ongoing transmission corresponding to level 5. A higher threat level indicates tougher social distancing and lockdown measures. The changes in the threat levels will be made based on medical and scientific data, such as the number of cases, the R_0_, and transmission rate, and will be operated by the Joint Biosecurity Centre in collaboration with the government. This system is expected to facilitate a relatively smooth and efficient transition between threat levels. When the system launched on May 10, 2020, the United Kingdom was declared to be in level 4 (severe), which denotes partial lockdown measures, ongoing community transmission, and slightly stretched yet coping health care systems [69].

Compared to New Zealand and the UK systems mentioned, the prototype model we propose has several advantages. First, we adopted precautionary measures to define the threat levels rather than the less descriptive terms used in the existing systems. For example, if a threat level is labeled as “severe” or “restrict,” it would not be readily understandable by the public unless they thoroughly study the alert system. The use of the terminology “prepare,” “reduce,” and “restrict” could also confuse people about which levels are being referred to if these are not accompanied by the numerical threat levels. The terms “substantial,” “severe,” and “critical” could mean the same intensity to the public and, therefore, always need to be followed by the level number when mentioned. In contrast, the use of precautionary measures as a level description is self-explanatory because common people understand the meaning without any additional effort. For example, if we say that the “Strict Physical Distancing” level is in effect, people would be able to visualize at which point they are in the hierarchy of the notification system. Second, the use of one logo per level could have an additional benefit, especially for people who are illiterate, as they can readily comprehend it by observing the logo. Third, as these terms of precautionary measures have become universal because of the current pandemic, any visitor in the foreign county would also be able to understand the prevention measures they must abide by for the current threat level in effect.

Aside from these newly implemented alert systems, sporadic use of the emergency notification systems has also been observed. In the province of Ontario, Canada, emergency alerts have been dispatched to inform dwellers about self-isolation and stay-home orders [71]. In addition, the use of existing disaster alert systems for the purpose of the COVID-19 pandemic was also evident. For example, Alert Bay, a remote coastal British Columbia community, has been using a tsunami warning siren to inform the residents about the COVID-19 curfew in the locality [72]. However, there is still a lack of a coordinated and systematic approach, which hinders the efficiency of the system and the effectiveness of the control measures [73]. This increases the possibility that critical recommendations, modifications, and warnings would be overlooked, especially for those who are not actively looking for the information. It might be a contributing factor that many residents ignored stay-home orders at the beginning [74]. Therefore, only imposing preventive measures would not be conducive unless citizens, for whom such measures are intended, are informed proactively. A recent survey by Calgary-based Public Emergency Alerting Services Inc is worth noting, which reported that about 84% of Canadians believe that the public should be informed about COVID-19 situations through the national public alert system [75]. It signifies the fact that citizens wish to be advised by the official sources in the event of a national crisis, at least in Canada.

Therefore, in similar developed countries where mobile phone penetration is high and wherein the trust of government is at least moderately high, a graded pandemic notification system may be a potential advantage over the status quo. The efficiency of the proposed notification system, however, would largely depend on the adherence and compliance of the citizens to the system, and how promptly the system could be activated. For instance, a measure like physical distancing would be most effective in containing an epidemic only if the public strictly complies with it and commences at the onset of the epidemic [76-79]. An organized, systematic, and coordinated approach is needed to receive the ultimate benefit from the system. The incorporation of legal enforcement could be necessary for the optimal functioning of the system, and the precautionary measures related to the threat levels should be prompt upon notification. To illustrate, when a lower threat level is expected and dispatched (level 1-2), the public would efficiently follow general or enhanced hygiene as instructed. Nonetheless, for the higher threat levels (level 3-5), additional safety measures would be expected by law and subject to sanction for noncompliance. The sanction could be a monetary penalty or other strict sanction proportionate to the severity of violation [80].

All institutions and organizations would need to develop their own logistics so that they can comply with the systems effectively upon notification. For example, when a level 4 (shelter in place) alert will be dispatched, the employees would start working from home where applicable, nonessential business and schools would be closed, and people would not leave home except for the necessary activities (among other measures) until the threat level is reduced. A change in the alert level would ideally be made either in the early morning or late evening or night to prevent road congestion and panic buying. Overall, installing such a system would operate complementary to any existing platform, and a significant benefit can be conferred by enhanced public engagement.

Given that bidirectional oscillations of threat levels over the course of a pandemic may be the norm, the proposed notification system may facilitate population response changes in the direction of increasing or decreasing precautions over time as the threat accumulates or abates. In all instances, such oscillations in response recommendation should be guided first and foremost by objective scientific advice on level of health threat rather than political objectives or other nonhealth-related agendas.

## Unique Challenges for Developing Countries

The administration of the graded pandemic notification system would be more challenging in developing countries for several reasons [81]. First, the accessibility of the system could be an issue due to the lack of some infrastructure components, leading to asymmetries in the reach of warnings, which may amplify the exposure and structural response disparities. Most developing countries now have well-developed mobile network systems with a majority of the population having access to cell phones, but this issue would be an ongoing consideration. As an illustration, Bangladesh, a developing country in South Asia and home of more than 164 million people [82], has reported approximately 166 million mobile phone subscribers in February 2020 [83]. Other South Asian countries have a similar level of mobile phone penetration. India is estimated to have over 800 million mobile phone users for a population of more than 1.3 billion [82,84].

Although cell phones are ubiquitous and widespread, a large proportion of people do not have reliable data plans [85]. Consequently, a notification system may need to largely rely on simple text messaging to ensure it can reach the majority of the population. Contrary to Canada where we have Alert Ready [86], mobile phone carriers in some countries do not provide the government with channels to notify the population in cases of emergency. Some countries might not have adequate technology to provide emergency alerts. Deploying such systems in developing regions, therefore, might require changes to the current infrastructure to allow for such channels to be implemented. In addition, close collaborations with cell phone providers and public health officials are necessary for the successful implementation of such technological ecosystems.

Second, the nature of each precaution level in the notification system would have to be customized to the context of developing countries [81]. For instance, the ability of large swaths of the population to enact social distancing is questionable in some countries, simply based on population density. Bangladesh and India have a population density of 1116 and 420 per square kilometer, respectively, compared to only 4 and 35 per square kilometer for Canada and the United States [82]. Therefore, a 2-meter physical distancing guideline recommended in the ongoing COVID-19 outbreak is likely not achievable.

In a similar manner, developing countries should be judicious while imposing extreme measures like lockdowns because a large proportion of the population lives under the poverty line, and the governments have limited capacity to support their citizens amid lockdown. The recent lockdown in India is worth noting as the lives of migrant workers were under threat during their efforts to return home [87]. The creation of roadway congestion could also increase the risk of disease transmission [88].

Third, the effectiveness of the notification system could be hampered by conventional wisdom or mistrust of the government. For example, a widely held belief in Mexico is that people from low socioeconomic neighborhoods benefit from greater disease immunity because they are exposed to poor sanitation standards. This belief led the governor of the Mexican State of Puebla to recently state that poor people are immune to COVID-19 [89], contrary to the fact that low standards of living are strongly associated with impaired immunity from multiple routes (eg, malnutrition, chronic stress, unhealthy behavior), leading to a greater likelihood of morbidity and mortality from COVID-19 [90-93]. The effectiveness of any notification system is prefaced on the existence of belief systems that will make the public receptive to the messages being transmitted through the system. Mistrust of government, perhaps fostered by histories of institutionalized corruption or cronyism, may also predispose the public to ignore government warnings. In Latin America, such mistrust has led citizens to look to international sources for information on COVID-19 [94], thereby suggesting that government-sponsored apps may be disregarded altogether.

Fourth, the lessons from existing alert systems should be scrutinized. For instance, Bangladesh has been operating their Cyclone Warning System in coastal areas for decades in collaboration with the international development partners and nongovernmental organizations. This system has been successful in reducing human casualties and property damage [95,96]. However, it has been reported that a large percentage of the residents do not follow evacuation orders and do not take necessary protective measures even after receiving warning messages and being informed about the potential consequences of not evacuating [97]. The primary reasons behind this are mistrust in the warning message and failure to understand the instructions conveyed in them [97]. Nationwide implementation of a pandemic notification system, therefore, needs to address these issues carefully. The message conveyed must be simple and concise, and will need to be tailored according to the needs of the target population. To improve compliance and penetration, the simultaneous use of other means must be considered, such as announcements from churches or prayer houses, radio, television, newspaper, and other community sources.

## Source and Level Considerations

An important consideration in the implementation of any pandemic notification system would be the issuing source. In this viewpoint, we have assumed that a government agency is the primary source, given the use of reserved mobile communication channels and universal population reach within a geographic region. However, it is also possible that arms-length public health organizations within or outside of governments could be considered as a notification issuing source as well. In the COVID-19 pandemic response, many central government bodies responded in a delayed manner, which would not be helped by a notification system reliant upon their approval. On the other hand, it is difficult to envision a pandemic notification system that is permitted to operate without a substantial degree of direct input from an official government source. Ultimately along these lines, a pandemic response system that is synchronized with others around the world may be ideal, particularly given the potential for rapid spread of any contagious epidemic due to air travel. Yet, on the level of any individual country or subregion within a country, substantial negotiation and multilateral approval would be required for it to operate in a manner that could reach the population with uniformity.

A final consideration is the level at which the communication is made: national, provincial or state, or regional. Given that both outbreaks and recovery rates vary significantly by subregions within a country, an argument could be made that notifications should be delivered on that level. In some countries, such geographic units have all or most of the authority to introduce such communications and make provisions to handle changes in threat level. All of these issues must be considered on a country-by-country basis. In all cases, communications must prioritize population health first and foremost.

## Limitations

The main limitation of a pandemic notification system would be the unintended consequences that could occur as a result of adverse public reactions. However, this issue could be mitigated by proper education and building trust in the government. Governments, for their part, need to ensure support for all citizens to follow any recommended precautionary measures. An additional limitation would be the lack of sensitivity to local context when deployed at the national level. This is particularly important when restrictions ease if the rate of easing is different within different regions. Several other limitations have been pointed out in previous sections (eg, privacy concerns, inadequate technology, noncompliance, mistrust); however, a detailed discussion of each point is beyond the scope of this paper.

To our knowledge, this is the first paper to describe the benefits and challenges of an individual-level pandemic notification system delivered via mobile technology. We illustrated the context of why such a system is necessary and described a simple prototype model, its prospects, and potential barriers regarding implementation. However, there are other challenges that need to be addressed with the benefit of empirical data gleaned from population attitudinal surveys and real world implementational trials.

## Conclusion

Following SARS-CoV and MERS-CoV, another novel coronavirus (SARS-CoV-2) emerged and rapidly spread across the world. Despite having global public health surveillance systems (eg, ProMED, GPHIN) and their timely notification on COVID-19, many nations were unsuccessful in controlling the virus effectively. To prevent or slow the progression of future pandemics, we need a remolded system capable of facilitating effective communication and prompt activation of preventive measures. A graded, individual-level pandemic notification system could be an effective tool if customized and carefully implemented on the level of the individual country. In this system, the threat levels would be arranged hierarchically with relevant logos and a set of recommended precautionary measures for each level, which would be easily comprehensible to all citizens. Notifications provided by such a system could facilitate a prompt, timely, and coordinated response from all levels of governments and, most importantly, from the public. However, extensive preparation, advance education, and coordination by the government with all the relevant sectors involved in the dissemination of the alert are required to prevent unintended consequences. Public education and awareness are of utmost importance to ensure that the alert is understood and appreciated properly upon delivery. Under such circumstances, citizens would be able to act promptly in an organized manner relevant to the alert level without having unproductive panic and fear. It is worth noting that different countries and regions have their own limitations and challenges. Implementing a pandemic notification system would not be effective unless authorities address unique challenges in the local context. Particularly for the developing regions, careful consideration of limitations and potential unintended consequences is required.

## Abbreviations

COVID-19: coronavirus disease
GPHIN: Global Public Health Intelligence Network
MERS: Middle East respiratory syndrome
MERS-CoV: Middle East respiratory syndrome–related coronavirus
ProMED: Program for Monitoring Emerging Diseases
R_0_: basic reproduction number
SARS: severe acute respiratory syndrome
SARS-CoV: severe acute respiratory syndrome–related coronavirus
SARS-CoV-2: severe acute respiratory syndrome coronavirus 2
WHO: World Health Organization
