# Correction: Considerations for an Individual-Level Population Notification System for Pandemic Response: A Review and Prototype

**DOI:** 10.2196/21634

**Published:** 2020-06-22

**Authors:** Mohammad Nazmus Sakib, Zahid A Butt, Plinio Pelegrini Morita, Mark Oremus, Geoffrey T Fong, Peter A Hall

**Affiliations:** 1 School of Public Health and Health Systems University of Waterloo Waterloo, ON Canada; 2 Research Institute for Aging University of Waterloo and Schlegel Villages Waterloo, ON Canada; 3 Department of Systems Design Engineering University of Waterloo Waterloo, ON Canada; 4 eHealth Innovation Techna Institute University Health Network Toronto, ON Canada; 5 Institute of Health Policy, Management, and Evaluation Dalla Lana School of Public Health University of Toronto Toronto, ON Canada; 6 Department of Psychology University of Waterloo Waterloo, ON Canada; 7 Ontario Institute for Cancer Research Toronto, ON Canada

In “Considerations for an Individual-Level Population Notification System for Pandemic Response: A Review and Prototype” (J Med Internet Res 2020;22(6):e19930), a correction request received from the authors during proofreading was inadvertently missed by JMIR Publications staff before publication.

The first sentence of the Introduction section, the word “organisms” should have been corrected to “pathogens.” This text has now been corrected from:

“Despite a dramatic improvement in health care affordances and extensive public health measures around the world, the emergence and re-emergence of infectious organisms and associated diseases have become a common phenomenon.”

to:

“Despite a dramatic improvement in health care affordances and extensive public health measures around the world, the emergence and re-emergence of infectious pathogens and associated diseases have become a common phenomenon.”

The correction will appear in the online version of the paper on the JMIR Publications website on June 22, 2020, together with the publication of this correction notice. Because this was made after submission to PubMed, PubMed Central, and other full-text repositories, the corrected article has also been resubmitted to those repositories

